# Massive hemothorax secondary to internal jugular vein central venous catheter placement in a patient undergoing spinal surgery complicated by chest trauma: a case report

**DOI:** 10.1186/s13019-023-02194-5

**Published:** 2023-04-06

**Authors:** Tao-wu Gong, Yu-hang Zhu, Peng-cheng Zhao, Fan Zhang

**Affiliations:** Department of Anesthesiology, Affiliated Hospital of Zunyi Medical University, Zunyi, Guizhou China

**Keywords:** Internal jugular vein puncture, Multiple rib fractures, Central venous catheterization, Spinal surgery, Case report

## Abstract

**Background:**

Placement of a central venous catheter (CVC) is a common procedure for spinal surgery and is relatively safe under ultrasound guidance.

**Case presentation:**

We report the case of a 56-year-old female who underwent ultrasound-guided placement of an internal jugular vein CVC for fluid replacement during spinal surgery for thoracic vertebral burst compression fracture and multiple rib fractures as a result of a high-altitude fall injury. Hemothorax developed intraoperatively. During a thoracotomy, the tip of the CVC was found within the chest cavity. The presence of chest trauma may impact on clinician’s appreciation of the potential complications of internal jugular vein CVC placement.

**Conclusion:**

The present case demonstrates the need for clinical awareness of the potential complications of CVC placement in patients with chest trauma and the need for adequate training in this technique.

**Supplementary Information:**

The online version contains supplementary material available at 10.1186/s13019-023-02194-5.

## Background

Spinal surgery is characterized by a high degree of trauma, long duration, and substantial intraoperative blood loss [1]. Therefore, for patients with large trauma, rapid fluid replacement and central venous pressure (CVP) monitoring are required during spinal surgery. In comparison with traditional fluid replacement methods, the placement of a central venous catheter (CVC) has become the preferred choice in patients undergoing trauma surgery due to increased safety [2]. CVC placement is predominantly performed using the subclavian, internal jugular, or femoral veins [3]. Subclavian venipuncture has a high incidence of pneumothorax and femoral venous puncture is associated with an increased risk of infection [4, 5]. Consequently, the risk of these complications can be reduced by placing a CVC in the internal jugular vein. Therefore, despite life-threatening complications such as pneumothorax, hemothorax, and injury to large vessels, internal jugular vein CVC placement has obvious advantages in many clinical applications and is often preferred by clinicians [6]. Hemothorax is also a common complication of closed chest trauma [7]. Approximately 300,000 cases of traumatic hemothorax occur in the United States each year [8]. Accordingly, determining the cause of hemothorax is difficult when a jugular vein puncture is performed in cases of closed chest trauma. For the first time, we report a case of massive hemothorax following the placement of a CVC into the right internal jugular vein in a patient with spinal trauma complicated by closed chest trauma.

## Case presentation

We report the case of a 56-year-old female patient weighing 58 kg who was admitted to the emergency department of our hospital due to chest and back pain caused by injury following a fall from height. The present study was approved by the Ethics Committee of the Affiliated Hospital of Zunyi Medical University (KLL-2022-663).

Vital signs were stable. Relevant examinations were completed after admission. Blood routine examination demonstrated a hemoglobin (HB) level of 83 g/L, red blood cell (RBC)-specific volume of 0.26, and an RBC count of 2.68 × 10^12^/L. Chest computed tomography (CT) revealed a T4 vertebral burst compression fracture, suspected spinal cord injury, and fractures of the first to third right ribs and the first to fifth left ribs. Closed thoracic drainage was not performed. Internal fixation of the T4 vertebral fracture via a posterior approach under general anesthesia was proposed.

Placement of a right internal jugular vein CVC and arterial line were performed under general anesthesia. Ultrasound of the right internal jugular vein revealed no abnormalities. The leading edge of the midpoint of the right sternocleidomastoid muscle is used as the puncture point. Ultrasound-guided down-plane puncture was performed, and the guide wire was successfully inserted and confirmed to be within the internal jugular vein using ultrasonography. A CVC was then placed after dilatation and confirmation of venous blood flow through the insertion needle. The CVC depth was 12 cm. Unobstructed flow was observed after skin fixation.

Four hours after surgery in the prone position, the patient’s blood pressure continued to decline. No obvious bleeding from the surgical area was observed, and the airway pressure was not significantly increased. The volume of the autologous blood recovery tank was 600 mL. Blood gas analysis demonstrated a Hb level of 83 g/L. Rapid fluid and autologous blood infusions were immediately administered. After the administration of vasopressors, the systolic blood pressure was maintained at 85–90 mmHg. At 50 min postoperatively, the blood pressure gradually decreased again with a lowest invasive blood pressure (IBP) of 60/30 mmHg, heart rate of 140 bpm, SpO_2_ of 100%, and central venous pressure of 10 cmH_2_O. Blood gas analysis demonstrated an HB level of 76 g/L. Fluid resuscitation and the administration of vasopressors failed to improve hypotension. At that time, the operation was completed, and the patient was quickly placed in the supine position.

**Fig. 1:**
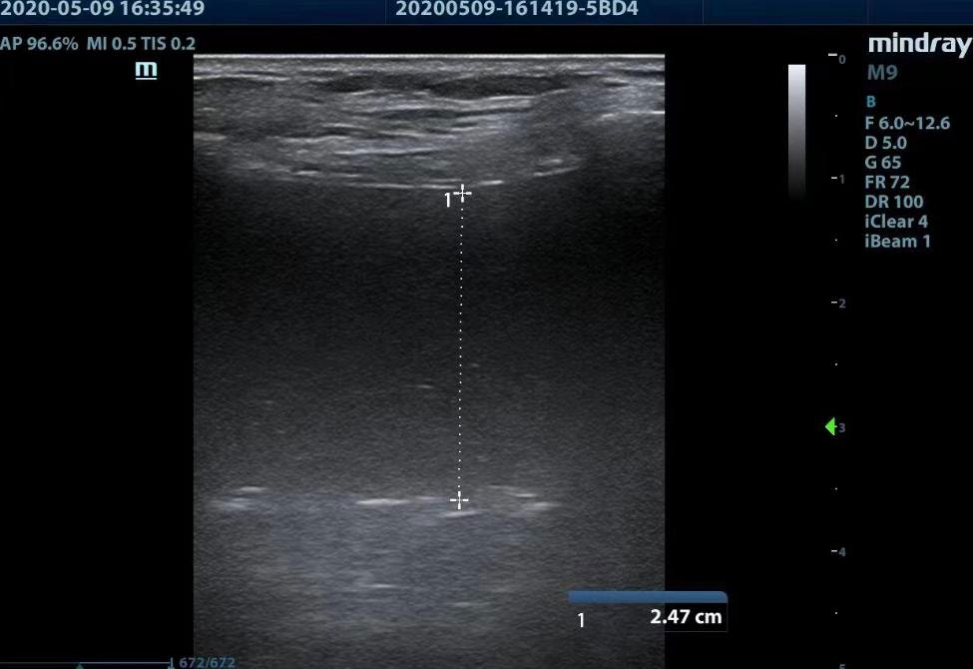
Ultrasonography demonstrating the presence of pleural effusion in the right midaxillary line

**Fig. 2:**
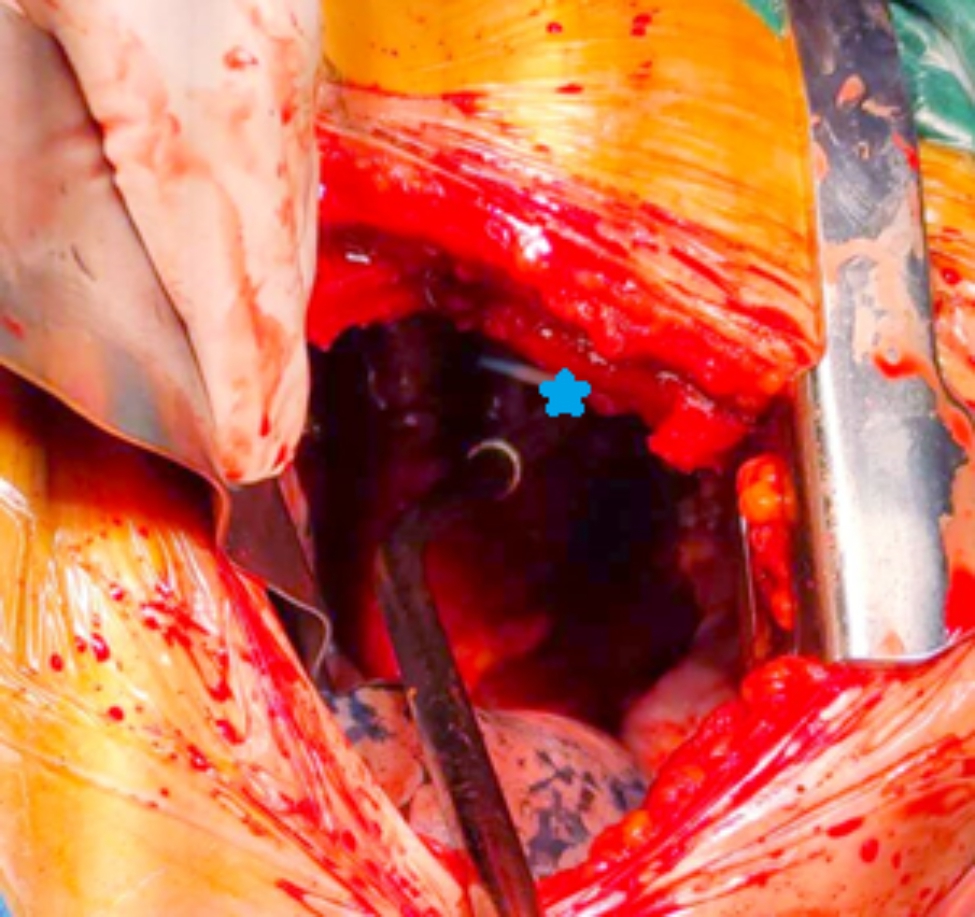
Internal jugular central venous catheter (pentagram) within the thorax

No pneumothorax sign was seen on ultrasonography of the second intercostal space; however, a large number of hypoechoic regions were observed in the midaxillary line and posterior axillary line (Fig. 1). Accordingly, hemothorax was considered. The thoracic surgery department was immediately consulted and closed thoracic drainage was performed. Approximately 1400 mL of fresh blood was slowly extracted in stages. At that time, the blood pressure had slightly recovered. After an interval of several minutes, the blood pressure significantly decreased again. A large volume of blood remained in the thoracic cavity that was amenable to drainage. After a multidisciplinary discussion, an explorative thoracotomy was performed. Thoracotomy revealed the CVC tip within the thoracic cavity (Fig. 2) and was considered to be the cause of massive hemorrhage into the thoracic cavity. Closed thoracic drainage was performed after further bleeding or injury had been excluded. After intraoperative treatment, the patient’s vital signs stabilized and he was admitted to our ICU. The tracheal tube was removed on the third day postoperatively, and the patient was discharged on postoperative day 18.

## Discussion

CVC placement is associated with a range of complications [3]. The development and application of ultrasound technology have improved the safety and quality of CVC placement [9]. In the present case, we report for the first time the development of large-volume hemothorax in a patient with spinal trauma complicated by chest trauma and multiple rib fractures after placement of an internal jugular vein CVC.

We initially considered two possible causes of hemothorax. The first was chest trauma with multiple rib fractures, which may have punctured the pleura and blood vessels resulting in hemothorax. The other was hemothorax secondary to CVC placement despite the substantial experience of the attending anesthesiologist in CVC placement and successful puncture confirmed via ultrasonography. Thoracotomy demonstrated the hemothorax in the present case was caused by internal jugular vein CVC placement. We speculate that deep insertion of the dilator or deep penetration of the guidewire front during dilator placement may have resulted in penetration of the pleural space. The incidence of hemothorax related to internal jugular vein CVC placement is reportedly low. Therefore, this complication can be overlooked when selecting an appropriate site for CVC placement, particularly in patients with chest trauma which is more likely to interfere with the clinical judgment regarding the potential complications of internal jugular vein CVC placement.

The majority of complications of CVC placement within the internal jugular vein are related to punctures in the short axis or outside the plane on ultrasonography leading to poor visualization of the tip of the puncture needle. The findings in the present case highlight the need for improved clinician awareness of the potential complications of CVC placement and the importance of training in this technique. Training in standardized ultrasonography and CVC placement techniques can improve the clinical safety and quality of CVC placement, thereby reducing the risk of associated complications [10].

## Electronic supplementary material

Below is the link to the electronic supplementary material.

Additional File: Plagiarism report

## Data Availability

All data generated or analyzed during this study are included in this published article. Data sharing is not applicable to this article as no datasets were generated or analyzed during the present study.

## Abbreviations

CVP: Central venous pressure
RBC: Red blood cell count
CT: Computed tomography
IBP: Invasive blood pressure
