# Supplementary material for: Massive hemothorax secondary to internal jugular vein central venous catheter placement in a patient undergoing spinal surgery complicated by chest trauma: a case report
Source: J Cardiothorac Surg. 2023 Apr 6;18:104. doi: 10.1186/s13019-023-02194-5 (PMC10080942; doi:10.1186/s13019-023-02194-5)
Supplement: Supplementary file 1 — Additional File: Plagiarism report [file 13019_2023_2194_MOESM1_ESM.pdf]

:enago

Author First, Quality First

www.enago.cn

CERTIFICATE OF EDITING

This is to certify that the paper titled Massive hemothorax secondary to internal jugular vein central venous catheter placement in a patient undergoing spinal surgery complicated by chest trauma: a case report commissioned to us by Taowu Gong has been edited for English language, grammar, punctuation, and spelling by Enago, the editing brand of Crimson Interactive Consulting Co., Ltd..

ISO 17100:2015
Translation Service Providers

ISO 27001:2013
Information Security Management System

ISO 9001:2015
Quality Management System

Issued by:

Enago, Crimson Interactive (Beijing) Consulting Co., Ltd.
Room 3217, Cyber Tower A, No. 2,
Zhongguancun South Street,
Haidian District, Beijing

Disclaimer: The intent of the author's message has been preserved during the editing process. The author is free to accept or reject our changes in the document after reviewing our editing. This certificate has been awarded at the time of sharing the final edited version (full file or sections of the file) with the author. Enago does not bear any responsibility for any alterations done by the author to the edited document post 20th Dec. 2022.

Japan	www.enago.jp, www.ulatus.jp, www.voxtab.jp	Russia	www.enago.ru
Taiwan	www.enago.tw, www.ulatus.tw	Arabic	www.enago.ae
China	www.enago.cn, www.ulatus.cn	Turkey	www.enago.com.tr
Brazil	www.enago.com.br, www.ulatus.com.br	S. Korea	www.enago.co.kr
Germany	www.enago.de	Global	www.enago.com, www.ulatus.com, www.voxtab.com

About Crimson:
Crimson Interactive Consulting Co. Ltd. is one of the world's leading academic research support services. Since 2005, we’ve supported over 2 million researchers in 125 countries with their publication goals.
